# Hypoxia‐inducible factor 1‐alpha does not regulate osteoclastogenesis but enhances bone resorption activity via prolyl‐4‐hydroxylase 2

**DOI:** 10.1002/path.4906

**Published:** 2017-05-29

**Authors:** Philippa A Hulley, Tammie Bishop, Aude Vernet, Jurgen E Schneider, James R Edwards, Nick A Athanasou, Helen J Knowles

**Affiliations:** ^1^ Nuffield Department of Orthopaedics Rheumatology & Musculoskeletal Sciences University of Oxford Oxford UK; ^2^ Nuffield Department of Medicine University of Oxford Oxford UK; ^3^ BHF Experimental MR Unit University of Oxford Oxford UK; ^4^ Nuffield Department of Orthopaedics Rheumatology & Musculoskeletal Sciences, Nuffield Orthopaedic Centre University of Oxford Oxford UK

**Keywords:** osteoclast, hypoxia‐inducible factor (HIF), differentiation, bone resorption, PHD2

## Abstract

Osteogenic–angiogenic coupling is promoted by the hypoxia‐inducible factor 1‐alpha (HIF‐1α) transcription factor, provoking interest in HIF activation as a therapeutic strategy to improve osteoblast mineralization and treat pathological osteolysis. However, HIF also enhances the bone‐resorbing activity of mature osteoclasts. It is therefore essential to determine the full effect(s) of HIF on both the formation and the bone‐resorbing function of osteoclasts in order to understand how they might respond to such a strategy. Expression of HIF‐1α mRNA and protein increased during osteoclast differentiation from CD14+ monocytic precursors, additionally inducing expression of the HIF‐regulated glycolytic enzymes. However, HIF‐1α siRNA only moderately affected osteoclast differentiation, accelerating fusion of precursor cells. HIF induction by inhibition of the regulatory prolyl‐4‐hydroxylase (PHD) enzymes reduced osteoclastogenesis, but was confirmed to enhance bone resorption by mature osteoclasts. Phd2 ^+/−^ murine osteoclasts also exhibited enhanced bone resorption, associated with increased expression of resorption‐associated Acp5, in comparison with wild‐type cells from littermate controls. Phd3 ^−/−^ bone marrow precursors displayed accelerated early fusion, mirroring results with HIF‐1α siRNA. In vivo, Phd2 ^+/−^ and Phd3 ^−/−^ mice exhibited reduced trabecular bone mass, associated with reduced mineralization by Phd2 ^+/−^ osteoblasts. These data indicate that HIF predominantly functions as a regulator of osteoclast‐mediated bone resorption, with little effect on osteoclast differentiation. Inhibition of HIF might therefore represent an alternative strategy to treat diseases characterized by pathological levels of osteolysis. © 2017 The Authors. *The Journal of Pathology* published by John Wiley & Sons Ltd on behalf of Pathological Society of Great Britain and Ireland.

## Introduction

Bone is continuously remodelled, initially during skeletal formation and then during development and in response to traumatic events such as bone fracture. Remodelling is regulated by the co‐ordinated actions of osteoclasts, which resorb bone, and osteoblasts, which form new mineralized bone. Pathological bone loss occurs when the homeostatic relationship between osteoblasts and osteoclasts is disturbed in favour of osteoclast overactivation or lack of balancing osteoblast activity 1, 2, 3.

Osteoclast and osteoblast function is regulated by the hypoxia‐inducible transcription factor, HIF. HIF is a heterodimer comprising an inducible alpha subunit (HIF‐1α, HIF‐2α) and a constitutively expressed beta subunit (HIF‐β/ARNT). Under standard growth conditions, HIF‐α is proteasomally degraded following post‐translational hydroxylation by a set of prolyl‐4‐hydroxylase enzymes (PHD1–3) that targets it for interaction with the von Hippel–Lindau protein (VHL). However, HIF‐α accumulates under conditions associated with either reduced PHD enzyme activity (for example, hypoxia 4, 5 or oncogenic mutation 6) or enhanced translation of HIF‐α such that it exceeds the substrate capacity of the PHD enzymes (for example, insulin 7 or hepatocyte growth factor (HGF) 8 exposure). Stabilized HIF‐α then translocates to the nucleus, dimerizes with HIF‐β, and binds the hypoxia‐response element (HRE) of HIF target genes to initiate transcription.

HIF plays a crucial role in stimulating angiogenesis and subsequent new bone formation. This osteogenic–angiogenic link was first described using mice with osteoblast‐specific deletions in either *Vhl*, so overexpressing HIF‐α, or *Hif1a* itself. It was shown that overexpression of HIF‐α stimulated expression of the pro‐angiogenic vascular endothelial growth factor (VEGF), leading to the formation of highly vascularized, dense trabecular bone. Deletion of either *Hif1a* or *Hif2a* reduced vascularization, although deletion of *Hif1a* had a more striking effect of reducing trabecular bone formation, due to additional direct effects on osteoblast proliferation 9, 10. Combined osteoblast‐specific deletion of *Phd2* with either *Phd1* and/or *Phd3* also increased trabecular bone formation. This was partly due to increased angiogenesis and partly due to an HIF‐dependent increase in the production of osteoprotegerin (OPG), leading to suppression of osteoclastogenesis 11.

Such studies raised interest in therapeutic strategies aiming to activate HIF to restore bone mass. HIF stabilization using PHD enzyme inhibitors increased vascularity and stimulated new bone formation, improving bone mineral density and bone strength in murine models of bone fracture 12, 13, 14, 15, distraction osteogenesis 16, and osteoporosis 17, 18.

The above studies focused on osteoblasts, but it is important to also consider the effects of HIF activation on osteoclast formation and function. Osteoclasts form by the fusion of CD14+ monocytic precursors, in the presence of macrophage colony‐stimulating factor (M‐CSF) and receptor activator of nuclear factor kappa B ligand (RANKL), to produce multi‐nucleated cells that resorb bone 19, 20. Hypoxia/reoxygenation enhances osteoclastogenesis 21, 22, 23, 24, 25, but there is little evidence of whether HIF affects the differentiation process. *Hif1a* mRNA expression increased during osteoclast formation from murine monocytes 26, but as HIFα is regulated at the level of protein stability, this is not indicative of HIF pathway activation. There are also few and contradictory data regarding how HIF manipulation affects osteoclast differentiation. Reduced *Phd2* transcription downstream of a *Fra‐2* mutation in mice produced long bones containing numerous giant osteoclasts that expressed HIF‐1α 27. However, genetic deletion of *Hif1a* in murine osteoclasts did not affect osteoclast differentiation either *in vitro* or *in vivo* 26, whereas transfection with constitutively active HIF‐1α inhibited osteoclast formation 28. Similarly, osteoclastogenesis studies utilizing PHD enzyme inhibitors are contradictory 28, 29, 30.

Hypoxic induction of bone resorption by mature osteoclasts is, however, HIF‐1α‐dependent 24. HIF‐1α stimulates secretion of the pro‐resorptive adipokine angiopoietin‐like 4 (ANGPTL4) 31 and regulates an osteoclast‐specific metabolic response to hypoxia necessary to maintain the high rates of ATP production required for bone resorption 32. Osteoclast‐specific inactivation of HIF‐1α antagonized osteoporotic bone loss in mice, suggesting that HIF‐1α also promotes osteoclast activation and bone loss *in vivo* 26.

Osteoclasts play a central role in pathological osteolysis. In light of the interest in HIF pathway activation as a therapeutic strategy to improve bone formation and/or reduce bone loss, it is essential that we also improve our understanding of the effect(s) of HIF on osteoclast formation and function.

## Materials and methods

### Osteoclast culture

Peripheral blood mononuclear cells were isolated from leucocyte cones (NHS Blood and Transplant) using Histopaque. Positively‐selected CD14+ monocytes were seeded onto dentine discs or plastic dishes in α‐MEM (without ribonucleosides/deoxyribonucleosides, containing 10% FBS, 2 mm l‐glutamine, 50 IU/ml penicillin, and 50 μg/ml streptomycin sulphate). Osteoclast differentiation was induced by supplementing with M‐CSF (25 ng/ml, R&D Systems, Abingdon, UK) and RANKL (35 ng/ml, produced in‐house) every 3–4 days for 9 days. Hypoxic exposure was at 2% O_2_, 5% CO_2_, balance N_2_ in a MiniGalaxy incubator (RS Biotech, Irvine, UK). This work was approved by the Oxford Clinical Research Ethics Committee (C01.071).

### Osteoclast assays

Formalin‐fixed cells were stained for tartrate‐resistant acid phosphatase (TRAP) using naphthol AS‐BI phosphate as a substrate with reaction of the product with Fast Violet B salt. Vitronectin receptor (VNR) was detected by immunocytochemistry using a CD51/61 monoclonal antibody (anti‐human clone 23C6, anti‐mouse clone RMV‐7, 1:400; Bio‐Rad, Oxford, UK). Multinucleated cells containing three or more nuclei were considered osteoclasts. Resorption of dentine discs was visualized by staining with 0.5% toluidine blue, following the removal of adherent cells by sonication. Dentine discs were photographed, resorption tracks highlighted, and the resorbed area was quantified using ImageJ. Release of C‐terminal products of type I collagen from dentine was assayed in tissue culture media by CTX‐I ELISA (Immunodiagnostic Systems, Tyne & Wear, UK).

### Reverse transcription–quantitative PCR (RT‐qPCR)

RNA was extracted in TRI reagent, then DNase‐treated and eluted using a Direct‐Zol RNA Miniprep kit (Zymo Research, Irvine, CA, USA). RNA was reverse‐transcribed and quantitative PCR was performed using Fast SYBR Green Master Mix (Applied Biosystems, Warrington, UK) in a Viia7 Real‐Time PCR system (Applied Biosystems). Human primers were pre‐validated Quantitect primers (Qiagen, Manchester, UK). Murine sequence primers were designed in‐house (see supplementary material, Table S1). Comparative quantification was performed, with target gene mRNA levels normalized to that for β‐actin (*ACTB/Actb*).

### Western blotting

Cells were homogenized in HIF lysis buffer (6.2 m urea, 10% glycerol, 5 mm dithiothreitol, 1% sodium dodecyl sulphate, protease inhibitors). Primary antibodies were against HIF‐1α (clone 54, 1:1000; BD Biosciences, Oxford, UK), GLUT1 (ab14683, 1:2500; Abcam, Cambridge, UK), LDHA (NBP1‐48336, 1:2000; Novus Biologicals, Cambridge, UK), and β‐tubulin (clone TUB2.1, 1:2500; Sigma‐Aldrich, Dorset, UK).

### Transfections

Cells were transfected with PGK HRE–firefly luciferase (a gift from Professor AL Harris, Oxford, UK) and pHRG–TK *Renilla* luciferase plasmids (Promega, Southampton, UK) using Lipofectamine 2000 (Invitrogen, Paisley, UK) and then lysed for detection of luciferase activity after 24 h. Luminescence was assayed using the Dual‐Luciferase Reporter Assay System (Promega), with firefly luciferase normalized to the *Renilla* transfection control. Cells were transfected with 50 nm siRNA targeting *HIF1α* or a *HIF1α* scrambled control using RNAiMAX (Invitrogen). Duplexes were removed after 4 h and osteoclasts cultured for a further 48 h prior to assay.

### Mouse details and ethical approval

All animal experiments were performed in accordance with and with the approval of the UK Home Office Animals (Scientific Procedures) Act 1986 and Local Ethical Review Procedures (University of Oxford Medical Sciences Division Ethical Review Committee). *Phd2* ^+/−^ mice (*n =* 3) 33, 34 were on a pure C57BL/6 genetic background; *Phd1* ^−/−^ (*n =* 5) 35 and *Phd3* ^−/−^ (*n =* 4) 36, 37 mice were on a mixed Swiss/129/SvEv genetic background. Female mice and wild‐type littermate controls were sacrificed by cervical dislocation at 25 weeks of age.

### Murine osteoclast, osteoblast, and adipocyte culture

Marrow cells were flushed from the right femur and tibia, washed, resuspended in complete α‐MEM (containing 10% FBS, 2 mm l‐glutamine, 50 IU/ ml penicillin, and 50 mg/ml streptomycin sulphate), and seeded into 24‐well plates at 5 × 10^5^ cells per well. After 2 h incubation, non‐adherent bone marrow cells were reseeded onto dentine discs or plastic dishes and treated with M‐CSF (25 ng/ml) and murine RANKL (50 ng/ml; Peprotech, London, UK) every 3–4 days for 9 days to induce osteoclast formation.

Adherent marrow cells were supplemented to encourage osteogenic differentiation (50 μg/ml ascorbic acid phosphate, β‐glycerophosphate 2 mM, dexamethasone 10 nm) or adipogenic differentiation (100 μm oleic acid). Media were changed every 3–4 days for 28 days. Osteoblast cultures were fixed on day 14 for alkaline phosphatase staining, using naphthol AS‐MX phosphate as a substrate and reaction of the product with Fast Violet B salt, and on day 28 for assessment of mineralization by staining with Alizarin Red S. Plates were scanned and the area and intensity of staining were quantified using ImageJ. Adipocyte cultures were also fixed on day 28 for assessment of triglyceride levels by staining with Oil‐Red‐O, which was extracted with isopropanol, and absorbance was measured at 510 nm.

### Micro‐CT and histomorphometry

The volume and architecture of trabecular bone in the distal femoral metaphysis and lumbar vertebrae (V2–V5) were analysed using a SkyScan 1172 micro‐CT scanner (SkyScan, Kontich, Belgium). Bones were fixed in 10% formalin and then wrapped in wet absorbent paper and Parafilm to prevent drying during scanning. The bones were scanned at an isotropic pixel size of 9.9 μm, with a voltage of 37 kV and a current of 228 mA, with a 0.5 mm Al filter and 450 projections. Images were reconstructed with NRecon software (SkyScan 1172). Fractional bone volume (BV/TV) and trabecular thickness (Tb.Th), spacing (Tb.Sp), and number (Tb.N) were calculated using Skyscan CT‐Analyser software. The tissue volume of interest was derived by generating a contour around the trabecular bone that excluded the cortical bone, starting at least 100 μm from the growth plate and extending for 900 μm (femur) or 650 μm (vertebrae).

Following micro‐CT, long bones and vertebrae were decalcified in 10% EDTA prior to wax embedding. Sections were cut at a depth so as to visualize the entire internal cross‐sectional area of the bone. Non‐serial sections were stained for H&E and with a post‐coupling staining technique to visualize TRAP, according to standard protocols. Cellular distribution within the metaphyseal region was quantified histomorphometrically using Osteomeasure quantification software (Osteometrics, Decatur, GA, USA).

### OPG and CTXI ELISA

Serum was collected from mice at sacrifice by cardiac puncture and stored at −80°C. Assay of the C‐terminal telopeptide of type I collagen (CTXI) as a bone resorption marker was performed using a Murine CrossLaps Serum EIA (IDS Systems, Tyne and Wear, UK). Serum levels of osteoprotegerin (OPG) were assessed using an OPG Quantikine ELISA (R&D Systems).

### Statistics

Results are derived from at least three independent experiments. Data are presented as mean ± standard error and were analysed using GraphPad Prism. Statistical analysis comprised two‐way or one‐way ANOVA using Dunnett's multiple comparison as a *post‐hoc* test, except for experiments with only two conditions, where a *t*‐test was applied. Results were considered significant at *p* < 0.05.

## Results

### HIF‐1α expression increases during osteoclast differentiation

**Figure 1:**
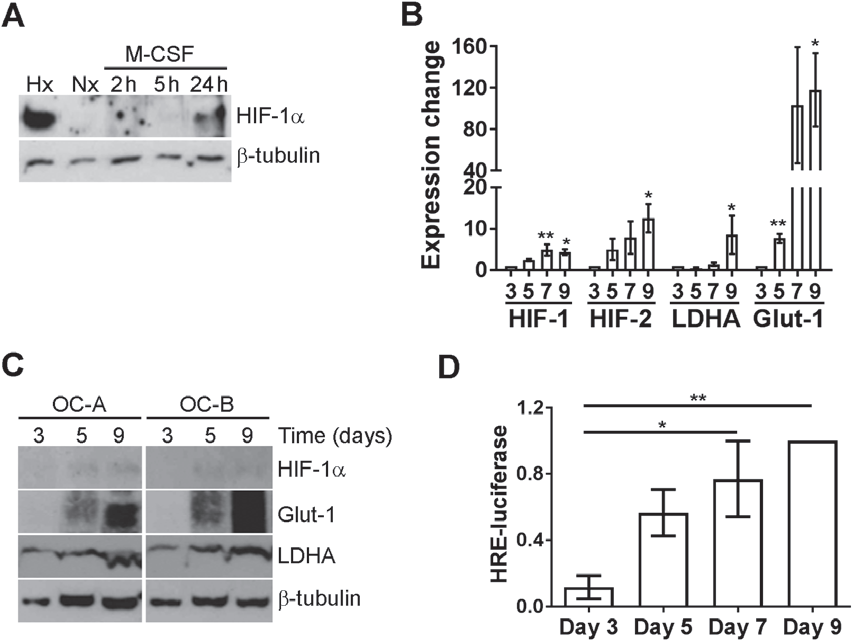
HIF induction during osteoclast differentiation. (A) Western blot analysis of HIF‐1α protein expression in CD14+ monocytes treated with 25 ng/ml M‐CSF for 2–24 h versus the untreated normoxic control (Nx). Hx = hypoxia (2% O_2_, 24 h). (B) RT‐qPCR comparing HIF1A, HIF2A, LDHA, and SLC2A1 (GLUT1) mRNA on days 3, 5, 7 and 9 of osteoclast differentiation. *p < 0.05; **p < 0.01. (C) Western blot analysis of HIF‐1α, GLUT1, and LDHA protein on days 3, 5, and 9 of osteoclast differentiation. (D) Normalized HRE‐driven luciferase reporter luminescence on days 3, 5, 7, and 9 of osteoclast differentiation. *p < 0.05; **p < 0.01.

Osteoclast differentiation from CD14+ monocytes requires M‐CSF and RANKL. We reported that M‐CSF stabilized HIF‐1α protein in mature human osteoclasts in normoxic culture 38. M‐CSF also stabilized HIF‐1α in normoxic CD14+ monocytes (Figure 1A), suggesting that HIF expression might increase throughout M‐CSF‐ and RANKL‐induced osteoclastogenesis. *HIF1A* and *HIF2A* mRNA expression increased during normoxic differentiation (Figure 1B), with HIF‐1α protein being detected from differentiation day 5 (Figure 1C). HIF‐2α protein was undetectable by western blot. HIF target gene expression (Figure 1B, C) and HRE‐driven luciferase production (Figure 1D) increased as differentiation progressed.

### HIF‐1α moderately inhibits cell fusion during osteoclastogenesis

**Figure 2:**
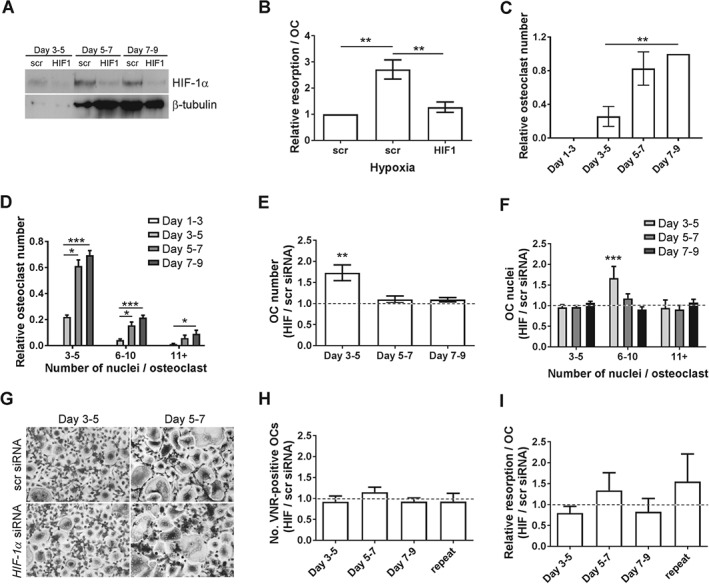
Effect of HIF1α siRNA on osteoclast differentiation. (A) Western blot analysis showing the efficacy of knockdown achieved by 48 h pre‐exposure to HIF1α siRNA compared with HIF1α scrambled (scr) control siRNA on days 5, 7, and 9 of osteoclast differentiation. HIF‐1α was stabilized with CoCl_2_ to facilitate visualization of the efficacy of HIF‐1α knockdown (CoCl_2_ was not used in the subsequent panels, which represent specific effects of the knockdown of differentiation‐induced HIF). (B) Relative resorption per osteoclast following treatment of mature (day 9) osteoclasts with HIF1α or control (scr) siRNA and exposure to hypoxia (2% O_2_) for 24 h. **p < 0.01. (C) Relative number of TRAP‐positive multi‐nucleated (≥3 nuclei) osteoclasts and (D) relative number of small (3–5 nuclei), medium (6–10 nuclei), and large (> 11 nuclei) osteoclasts formed on days 3, 5, 7, and 9 of monocyte–osteoclast differentiation following 48 h pretreatment with control (scr) siRNA. *p < 0.05; ***p < 0.001. (E) Relative number of TRAP‐positive multi‐nucleated (≥ 3 nuclei) cells formed on days 5, 7, and 9 of osteoclast differentiation following 48 h pretreatment with HIF1α siRNA, in comparison with control (scr) siRNA. (F) Relative number of small (3–5 nuclei), medium (6–10 nuclei), and large (> 11 nuclei) osteoclasts formed in E. **p < 0.01; ***p < 0.001. (G) Representative images of TRAP‐positive multi‐nucleated cells quantified in E and F. (H) Relative number of mature, VNR‐positive osteoclasts formed on day 9 and (I) resorption per osteoclast on day 9 following 48 h treatment with HIF1α siRNA from day 3, 5 or 7 of differentiation, in comparison with control (scr) siRNA. Repeat = siRNA treatment at each time point.

To determine whether differentiation‐induced HIF‐1α drives osteoclastogenesis, *HIF1α* siRNA was applied for 48 h during different stages of differentiation. *HIF1α* siRNA ablated HIF‐1α protein expression (Figure 2A) and functionally inhibited the hypoxia‐induced increase in bone resorption by mature osteoclasts (Figure 2B), as previously reported 24, 32. Cells treated with control siRNA successfully formed osteoclasts (Figure 2C), which increased in size as differentiation progressed (Figure 2D). Day 3 cells contained fewer than three nuclei and could not yet be considered osteoclasts. As HIF‐1α was undetectable on day 3 (Figure 1C), and as there were issues with transfection‐related toxicity in early monocytes, experimental *HIF1α* siRNA was started from differentiation day 3.

*HIF1α* siRNA treatment from day 3 to day 5 produced a 73.0 ± 18.6% (*p* < 0.005) increase in the number of osteoclasts formed by day 5 (Figure 2E), which were larger than cells treated with control siRNA (Figure 2F). *HIF1α* siRNA had no effect at later time points. When cells were allowed to differentiate to maturity following siRNA treatment, no effect of *HIF1α* siRNA was observed on osteoclast formation (Figure 2G) or on the final resorption activity (Figure 2H).

### HIF enhances resorption, but inhibits osteoclast differentiation

**Figure 3:**
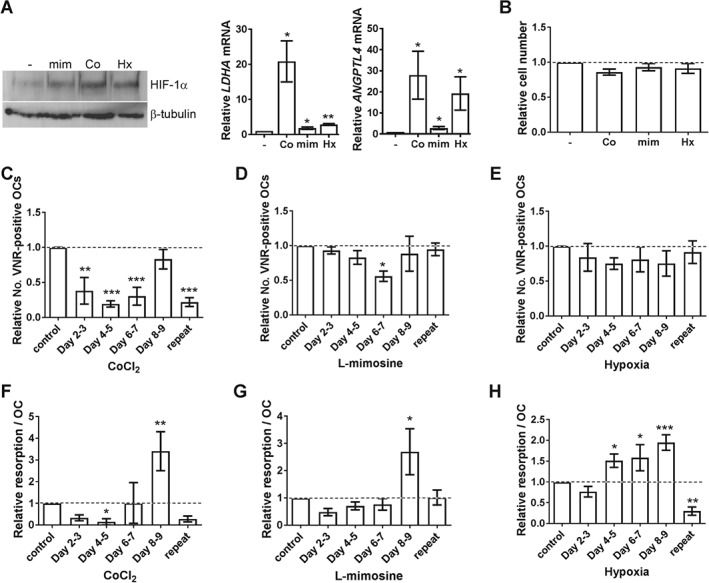
HIF induction inhibits osteoclast differentiation. (A) Western blot analysis of HIF‐1α protein expression in mature osteoclasts (day 9) following 24 h treatment with l‐mimosine (mim, 100 μm), CoCl_2_ (Co, 100 μm) or hypoxia (Hx, 2% O_2_). RT‐qPCR analysis of the same cells for expression of ANGPTL4 and LDHA mRNA. (B) Relative number of CD14+ monocytes, (C–E) relative number of VNR‐positive mature osteoclasts, and (F–H) relative amount of bone resorption per osteoclast following 24 h exposure to CoCl_2_ (Co, 100 μm), l‐mimosine (mim, 100 μm) or hypoxia (Hx, 2% O_2_) on days 2–3, 4–5, 6–7 or 8–9 of differentiation, or treatment at each time point (repeat). *p < 0.05; **p < 0.01; ***p < 0.001.

Next, we investigated whether HIF induction would affect osteoclast differentiation. Differentiation and the differentiation‐associated increase in HIF‐1α were induced with M‐CSF and RANKL and then HIF‐1α was further stabilized by exposure to CoCl_2_, l‐mimosine or hypoxia (2% O_2_) (Figure 3A). All stimuli were non‐toxic to CD14+ monocytes (Figure 3B) and mature osteoclasts (day 8–9 exposure; Figure 3C–E) and increased bone resorption by mature osteoclasts (Figure 3F–H). However, HIF induction during differentiation inhibited osteoclast formation on the scale CoCl_2_ > l‐mimosine > hypoxia, with CoCl_2_ dramatically reducing osteoclast numbers and hypoxia showing a general, but insignificant, dampening of osteoclast formation (Figure 3C–E). This produced a corresponding reduction in the final amount of resorption achieved (Figure 3F, G), except for hypoxia where early exposure consistently increased the final resorption capacity of the osteoclasts (Figure 3H).

### PHD2 depletion enhances osteoclast‐mediated bone resorption

These data suggest that HIF‐1α moderately delays monocyte–osteoclast differentiation, but substantially enhances osteoclast‐mediated resorption of bone. We therefore investigated the effects of genetic ablation of individual isoforms of the prolyl hydroxylase enzymes (PHD1–3). PHD2 is the main regulator of HIFα stability, PHD2 depletion being sufficient to stabilize HIFα and activate HIF‐mediated transcription 39. It is the only individual HIF hydroxylase with a reported bone phenotype 40, 41, 42, 43.

**Figure 4:**
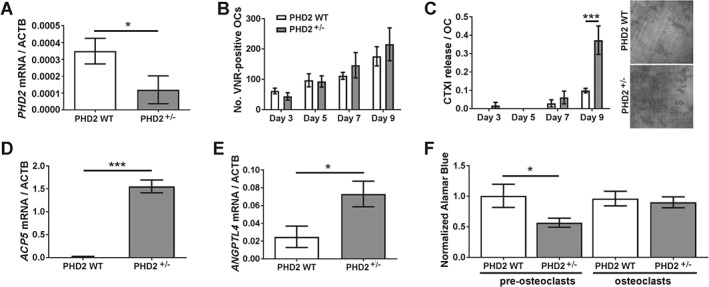
PHD2 regulates osteoclast bone resorption. (A) RT‐qPCR analysis of Phd2 mRNA expression in bone marrow cells from Phd2 ^+/−^ and Phd2 ^WT^ mice. *p < 0.05. (B) Number of VNR‐positive multi‐nucleated osteoclasts formed and (C) amount of resorption per osteoclast on days 3, 5, 7, and 9 of osteoclast differentiation from marrow‐derived Phd2 ^+/−^ and Phd2 ^WT^ precursors. ***p < 0.001. (D, E) RT‐qPCR analysis of (D) Acp5 and (E) Angptl4 mRNA in mature osteoclasts derived from Phd2 ^+/−^ and Phd2 ^WT^ marrow cells. *p < 0.05; ***p < 0.001. (F) Relative AlamarBlue® fluorescence per cell, as a readout of mitochondrial reductase activity, in bone marrow precursors and mature osteoclasts from Phd2 ^+/−^ and Phd2 ^WT^ mice. *p < 0.05.

Heterozygous depletion of *Phd2* was confirmed in bone marrow cells from *Phd2* ^*+/−*^ mice versus wild‐type controls (Figure 4A). No difference between *Phd2* ^*+/−*^ and *Phd2* ^*WT*^ marrow cells was observed in the rate of osteoclast formation, the number of osteoclasts formed (Figure 4B) or the expression of osteoclast differentiation‐related genes, e.g. *Nfatc1*, *Ctsk* (data not shown). However, mature *Phd2* ^*+/−*^ osteoclasts were 3.7 ± 0.8‐fold (*p* < 0.001) more resorptive than wild‐type osteoclasts (Figure 4C) and expressed more resorption‐associated *Acp5* (Figure 4D). They also showed increased expression of the pro‐resorptive HIF‐1α‐dependent adipokine *Angptl4* (Figure 4E) and an altered metabolic response to HIF. *Phd2* ^*+/−*^ bone marrow precursors exhibited reduced mitochondrial reductase activity as a result of the HIF‐mediated glycolytic switch. However, mature *Phd2* ^*+/−*^ osteoclasts differentiated from the same cell population maintained high levels of mitochondrial activity (Figure 4F), an osteoclast‐specific response to HIF necessary to maintain ATP production in hypoxia and support continued bone resorption 32.

### Reduced trabecular bone formation in Phd2 ^+/−^ mice

**Figure 5:**
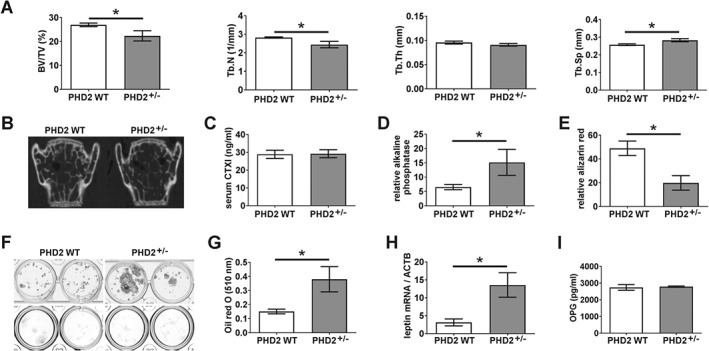
Bone formation in Phd2 ^+/−^ mice. (A) Comparison of vertebral BV/TV (%), Tb.N (1/mm), Tb.Th (mm), and Tb.Sp (mm) in Phd2 ^+/−^ and Phd2 ^WT^ mice. *p < 0.05. (B) Representative 2D image of vertebral bone from Phd2 ^+/−^ and Phd2 ^WT^ mice. (C) Serum levels of CTXI in Phd2 ^+/−^ and Phd2 ^WT^ mice. (D, E) Osteogenic differentiation of Phd2 ^+/−^ and Phd2 ^WT^ marrow cells showing (D) alkaline phosphatase activities on day 14 and (E) quantified alizarin red staining on day 28. *p < 0.05. (F) Representative plates showing osteogenic differentiation from n = 2 Phd2 ^+/−^ and Phd2 ^WT^ mice; alkaline phosphatase (top panel), alizarin red (bottom panel). (G, H) Adipogenic differentiation of Phd2 ^+/−^ and Phd2 ^WT^ marrow cells showing (G) quantified Oil Red‐O staining and (H) relative Lep mRNA levels on day 28. *p < 0.05. (I) Serum levels of OPG in Phd2 ^+/−^ and Phd2 ^WT^ mice.

We next studied the bone architecture of the *Phd2* ^*+/−*^ mice. Both *Phd2* ^*+/−*^ and *Phd2* ^*WT*^ mice had limited trabecular bone in the long bones (mean femoral BV/TV 2.48% ± 0.85), characteristic of their C57Bl/6 background 44. This prevented any differences in trabecular bone being observed, although an increase in cortical bone area was evident (supplementary material, Figure S1). We therefore studied the vertebrae and observed reduced BV/TV and trabecular number in the *Phd2* ^*+/−*^ mice, as well as increased trabecular spacing (Figure 5A, B). Vertebral histomorphometry detected no difference in the number of TRAP‐positive osteoclasts present, consistent with no effect of PHD2 on osteoclast formation. There was, however, also no difference in serum CTXI concentration (Figure 5C).

We therefore considered whether PHD2 depletion affects osteoblast mineralization. *Phd2* ^*+/−*^ marrow cells cultured in osteogenic media exhibited greater alkaline phosphatase activity, indicative of increased early osteoblast differentiation, but did not mineralize as well as *Phd2* ^*WT*^ cells (Figure 5D–F). Conversely, *Phd2* ^*+/−*^ marrow cells showed improved adipogenic differentiation, expressing 4.3‐fold more *Lep* mRNA than wild‐type controls (Figure 5G, H). However, no *in vivo* difference was observed in either osteoblast or adipocyte number (data not shown). Combined osteoblast‐specific knockdown of *Phd2* and *Phd3* was reported to stimulate OPG production and inhibit osteoclast activity 11. Serum levels of OPG were unaffected by depletion of *Phd2* alone (Figure 5I).

### PHD3 depletion moderately enhances early osteoclast differentiation

**Figure 6:**
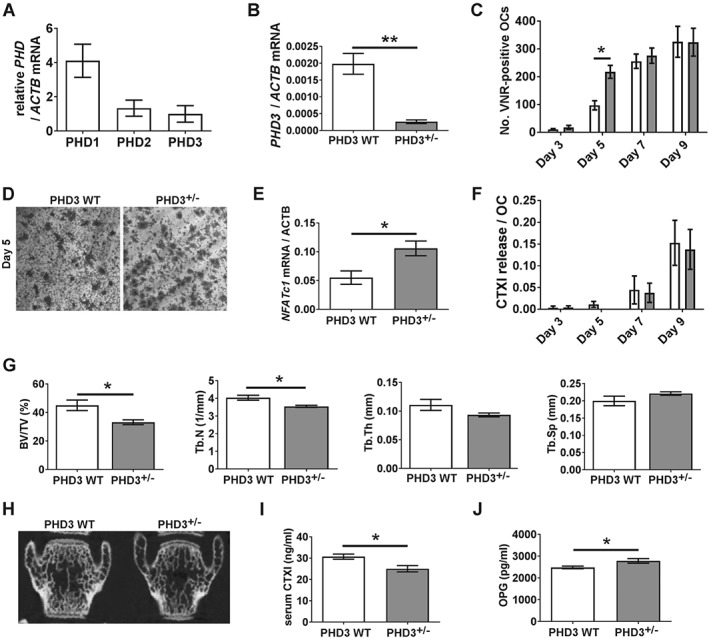
Osteoclast differentiation and bone formation in Phd3 ^−/−^ mice. (A) RT‐qPCR analysis of Phd1, Phd2, and Phd3 mRNA in osteoclasts derived from Swiss/129/SvEv wild‐type marrow cells.(B) RT‐qPCR analysis of Phd3 mRNA in bone marrow cells from Phd3 ^−/−^ and Phd3 ^WT^ mice. **p < 0.01. (C) Number of VNR‐positive multi‐nucleated osteoclasts formed on days 3, 5, 7, and 9 of osteoclast differentiation from marrow‐derived Phd3 ^−/−^ and Phd3 ^WT^ precursors. *p < 0.05. (D) Representative image of VNR‐positive osteoclasts formed on day 5 of differentiation. (E) RT‐qPCR analysis of Nfatc1 mRNA in marrow cells from Phd3 ^−/−^ and Phd3 ^WT^ mice. *p < 0.05. (F) Amount of resorption performed per osteoclast on days 3, 5, 7, and 9 of osteoclast differentiation from marrow‐derived Phd3 ^−/−^ and Phd3 ^WT^ precursors. (G) Comparison of vertebral BV/TV (%), Tb.N (1/mm), Tb.Th (mm), and Tb.Sp (mm) in Phd3 ^−/−^ and Phd3 ^WT^ mice. *p < 0.05. (H) Representative 2D image of vertebral bone from Phd3 ^−/−^ and Phd3 ^WT^ mice. (I, J) Serum levels of (I) CTXI and (J) OPG in Phd3 ^−/−^ and Phd3 ^WT^ mice. *p < 0.05.

We next investigated whether deletion of PHD1 or PHD3 would affect osteoclastogenesis and bone resorption. No difference was observed between *Phd1* ^*−/−*^ and *Phd1* ^*WT*^ cells in any parameter tested. This was not due to lack of *Phd1* mRNA; indeed, *Phd1* mRNA showed the greatest expression of all the PHDs in murine osteoclasts (Figure 6A).

Homozygous knockdown of *Phd3* was confirmed in bone marrow cells from *Phd3* ^*−/−*^ mice versus wild‐type controls (Figure 6B). *Phd3* ^*−/−*^ marrow cells formed osteoclasts more rapidly than wild‐type controls (Figure 6C, D), associated with increased expression of the early differentiation marker *Nfatc1* (Figure 6E). There was no difference between *Phd3* ^*−/−*^ and *Phd3* ^*WT*^ cells with respect to the final number of osteoclasts formed (Figure 6C) or their resorptive capacity (Figure 6F).

### Reduced trabecular bone formation in Phd3 ^−/−^ mice

We therefore studied the bone architecture of the *Phd3* ^*−/−*^ mice. Due to the higher trabecular bone content of Swiss/129/SvEv mice, we could detect a phenotype in both the long bones (supplementary material, Figure S2) and the vertebrae (Figure 6G, H), namely reduced BV/TV and trabecular number in the *Phd3* ^*−/−*^ mice and increased trabecular spacing. No cortical bone phenotype was evident (supplementary material, Figure S2). No *in vivo* difference in the number of TRAP‐positive osteoclasts was observed (data not shown). Intriguingly, serum CTXI levels were reduced in the *Phd3* ^*−/−*^ animals (Figure 6I).

We therefore investigated the effects of PHD3 depletion on osteoblastic and adipogenic differentiation. No differences were detected between *Phd3* ^*−/−*^ and *Phd3* ^*WT*^ cells in differentiation along either lineage or in osteoblast or adipocyte number by histomorphometry (data not shown). We did, however, detect increased serum concentrations of OPG in the *Phd3* ^*−/−*^ mice (Figure 6J).

## Discussion

We have demonstrated, for the first time, stabilization and transcriptional activation of HIF‐1α during osteoclastogenesis, although this was not required for differentiation. HIF activation by PHD enzyme inhibition suppressed osteoclast formation but enhanced osteoclast‐mediated bone resorption.

M‐CSF induced HIF‐1α expression in monocytes, as in osteoclasts 38. RANKL also induces *HIF1a* mRNA via activation of NF‐κB 26, 45. Both mechanisms likely contribute to the osteoclastogenic increase in HIF. It is likely that HIF also accumulates during osteoclastogenesis *in vivo*; we have previously described immunohistochemical expression of HIF‐1α in the mature osteoclasts present within osteolytic pathologies such as giant cell tumour of bone and rheumatoid arthritis 31, 38, 46. Osteoclastogenesis also induced HIF‐mediated transcription, as reported for the glycolytic enzymes and VEGF 45, 47, 48. Inhibiting HIF‐1α accumulation did not affect the final number of osteoclasts formed but accelerated cell fusion, a central feature of osteoclastogenesis. The effects of CoCl_2_ and l‐mimosine to inhibit osteoclast formation might be due to off‐target effects as they are not specific PHD enzyme inhibitors and will affect pathways besides those driven by HIF. It should be noted that HIF induced by exposure to hypoxia did not significantly affect differentiation.

We did not find that hypoxia/reoxygenation enhanced osteoclastogenesis, although our treatment schedule included more time in normoxia than generally reported 21, 22, 23, 24, 25. Given the moderately inhibitory effect of HIF‐1α on osteoclastogenesis, it is interesting to speculate whether it is reoxygenation, rather than hypoxia, that generally promotes osteoclast formation. Reoxygenation activates NF‐κB 49 and reactive oxygen species (ROS) 50, both of which promote osteoclast formation 51, 52, providing interesting candidates for further investigating this aspect of osteoclastogenesis.

It is intriguing that HIF enhances bone resorption by mature osteoclasts but moderately delays differentiation. HIF stimulates ANGPTL4 production, which promotes resorption without affecting differentiation 31. HIF also stimulates glycolysis 32, which drives bone resorption by mature osteoclasts 53, 54, 55. Osteoclasts micro‐compartmentalize glycolytic ATP generation at intracellular sites where it can directly support bone resorption, by promoting interaction between glycolytic enzymes and components of the resorption machinery 56, 57, 58. Conversely, energy for osteoclast differentiation derives from mitochondrial oxidative metabolism 47, 58, 59, 60. HIF‐mediated induction of glycolysis would therefore directly influence bone resorption without affecting differentiation.

This is the first description of direct effects of any of the PHD enzymes on osteoclasts. Increased osteoclast‐mediated bone resorption due to *Phd2* depletion was associated with increased *Acp5* expression. This was unexpected, as we have previously shown hypoxia to reduce *ACP5* mRNA expression 24. However, *ACP5* expression increases following prolonged hypoxic exposure 61 and hypoxia increases TRAP phosphatase activity 24.

Accelerated early osteoclast formation due to *Phd3* depletion was associated with increased expression of *Nfatc1*. NFATc1 is necessary for osteoclast differentiation 52 and is induced by hypoxia via inhibition of microRNA‐124 62 or PHD2 63. Accelerated early differentiation in *Phd3* ^*−/−*^ cells mirrored our results using *HIF1α* siRNA. *Phd3* depletion in the pre‐osteoclasts might produce a compensatory increase in the expression of *Phd2*, inhibiting HIF‐1α and stimulating early osteoclastogenesis. Alternatively, deletion of *Phd3* might prevent monocyte apoptosis and improve survival to promote early osteoclastogenesis 64.

Although increased bone resorption due to *Phd2* depletion was observed *in vitro*, there was no increase in serum CTXI concentration in the *Phd2* ^*+/−*^ mice, despite a reduction in trabecular bone. We consider that this might be due to the very low trabecular bone content of the *Phd2* ^*+/−*^ and *Phd2* ^*WT*^ mice, especially in the long bones, providing little surface area for osteoclasts to resorb. As osteoclast activity is also not constant, this could result in an undetectable difference in serum CTXI. Coupled with the long‐term effects of a reduction in osteoblast mineralization, these two effects would cause the diminished trabecular bone observed in the *Phd2* ^*+/−*^ mice.

It is interesting to compare the *Phd2* ^*+/−*^ bone phenotype with the conditional *Phd2* knockouts used in other musculoskeletal studies. Osteoblast‐specific deletion of *Phd2* caused reduced bone mass due to impaired osteoblast differentiation and mineralization 40, supporting *in vitro* data that hypoxia or PHD inhibition suppresses osteoblastogenesis 65, 66. Conditional deletion of *Phd2* in the monocyte/macrophage lineage caused reduced bone mass due to HIF‐mediated production of erythropoietin, which inhibited osteoblast mineralization and induced osteoclastogenesis and bone erosion 43. However, most *in vivo* work suggests that *Phd2* deletion increases bone mass. Osteoblast‐ 43 or chondrocyte‐specific 41 deletion of *Phd2*, or combined deletion of *Phd1–3* in osteoblasts 11, 67, increased trabecular bone mass. This was due to either enhanced osteoclast‐mediated bone resorption exceeded by increased bone formation 67, increased bone formation due to increased angiogenesis, or inhibition of osteoclastogenesis via HIF‐mediated up‐regulation of OPG 11.

Serum concentrations of OPG were elevated in our *Phd3* ^*−/−*^ mice, associated with reduced serum CTXI, indicative of reduced osteoclast activity *in vivo* due to inhibitory effects of osteoblast and/or stromal cell‐derived OPG. We speculate that the reduced bone mass phenotype of the *Phd3* ^*−/−*^ mice might therefore relate to a long‐term reduction in *in vivo* osteoblast mineralization. This could potentially be mediated by dysregulation of the sympathetic nervous system in the *Phd3* ^*−/−*^ animals 36, the function of which is critical for bone development and remodelling 68, or by inhibitory interactions with other musculoskeletal cell types or by endocrine signalling.

Combined PHD enzyme knockdown seemingly corroborates studies using PHD inhibitors to improve bone mass 12, 13, 14, 15, 16, 17, 18. Only one study mentions the *in vivo* osteoclast response to HIF activation. Dimethyl‐oxalyl‐glycine (DMOG) did not affect either the osteoporotic increase in osteoclast number or the increase in serum CTXI concentration in ovariectomized (OVX) mice, despite improvements in bone mass 18. This indicates that PHD enzyme inhibition and HIF activation *in vivo* promotes osteoblast‐mediated bone formation and overrides mechanisms to enhance osteoclast‐mediated resorption of bone. This suggests exciting opportunities for PHD inhibition in other osteolytic pathologies, such as rheumatoid arthritis.

The striking effect of HIF‐1α to enhance osteoclast‐mediated bone resorption alternatively suggests HIF inhibition as a strategy to treat osteolytic disease. Administration of the HIF inhibitor 2‐methoxyestradiol (2ME) protected OVX mice from osteoclast activation and bone loss 26 and reduced osteolysis in models of metastatic bone cancer 69, 70 and rheumatoid arthritis 71, 72. Although 2ME is not a specific inhibitor of HIF, these studies suggest this approach as a promising alternative therapeutic strategy.

In summary, the first direct comparison of the effects of HIF knockdown (*HIF1α* siRNA), HIF induction (hypoxia, CoCl_2_, l‐mimosine), and PHD depletion (*Phd1* ^*−/−*^, *Phd2* ^*+/−*^, *Phd3* ^*−/−*^) in osteoclasts suggests a striking role for HIF‐1α/PHD2 to drive bone resorption by mature osteoclasts, alongside a moderate effect of HIF to delay cell fusion during differentiation.

Constitutive PHD2 knockdown and activation of HIF‐mediated transcription *in vivo* produces a correspondingly low bone mass phenotype. HIF inhibitors might therefore represent an alternative therapeutic strategy to treat pathological osteolytic diseases including cancer, rheumatoid arthritis, osteoporosis, and bone fracture.

## Acknowledgements

We thank Douglas de Pasos Santos and Julie Adam for *in vivo* technical assistance, Dr Orla Gallagher and Dr Les Coulton for pilot micro‐CT work, and Professor Chris Pugh for helpful discussions. PH is funded by grants from UCB Pharma (R41281CN002), Nc3Rs (NC/L001403/1), and MRC/NIHR EME (12/206/30). TB is supported by the Wellcome Trust and the Ludwig Institute for Cancer Research. JS acknowledges funding from the Oxford BHF Centre for Research Excellence. JE is funded by grants from Arthritis Research UK (Fellowship 20631) and Orthopaedic Research UK (509). HK is funded by grants from Arthritis Research UK (Fellowship MP/19200) and The Rosetrees Trust (M456). Work in the Nuffield Department of Orthopaedics, Rheumatology and Musculoskeletal Sciences (NDORMS) is additionally supported by the Oxford National Institute of Health Research (NIHR) Musculoskeletal Biomedical Research Unit (BRU).

## Author contributions statement

PH and HK designed the study, performed experiments, and collected, analysed and interpreted the data. NA helped design the study. TB, AV, and JS performed experiments and collected data. JE acquired and interpreted the data. All authors were involved in writing the paper and had final approval of the submitted and published versions.

**SUPPLEMENTARY MATERIAL ONLINE**

**Figure S1.** Micro‐CT of femoral trabecular and cortical bone from *Phd2* ^*+/−*^ and *Phd2* ^*WT*^ mice

**Figure S2.** Micro‐CT of femoral trabecular and cortical bone from *Phd3* ^*−/−*^and *Phd3* ^*WT*^ mice

**Table S1.** List of primers used for RT‐qPCR

## Supporting information


**Figure S1.** MicroCT of femoral trabecular and cortical bone from Phd2 ^+/‐^ and Phd2 ^WT^ mice

**Figure S2.** MicroCT of femoral trabecular and cortical bone from Phd3 ^‐/‐^and Phd3 ^WT^ mice

**Table S1.** List of primers used for RT‐qPCR
