# Supplementary material for: Hypoxia‐inducible factor 1‐alpha does not regulate osteoclastogenesis but enhances bone resorption activity via prolyl‐4‐hydroxylase 2
Source: J Pathol. 2017 May 29;242(3):322–33. doi: 10.1002/path.4906 (PMC5518186; doi:10.1002/path.4906)
Supplement: Supplementary file 1 — Figure S1. MicroCT of femoral trabecular and cortical bone from Phd2 +/‐ and Phd2 WT mice [file PATH-242-322-s001.pdf]

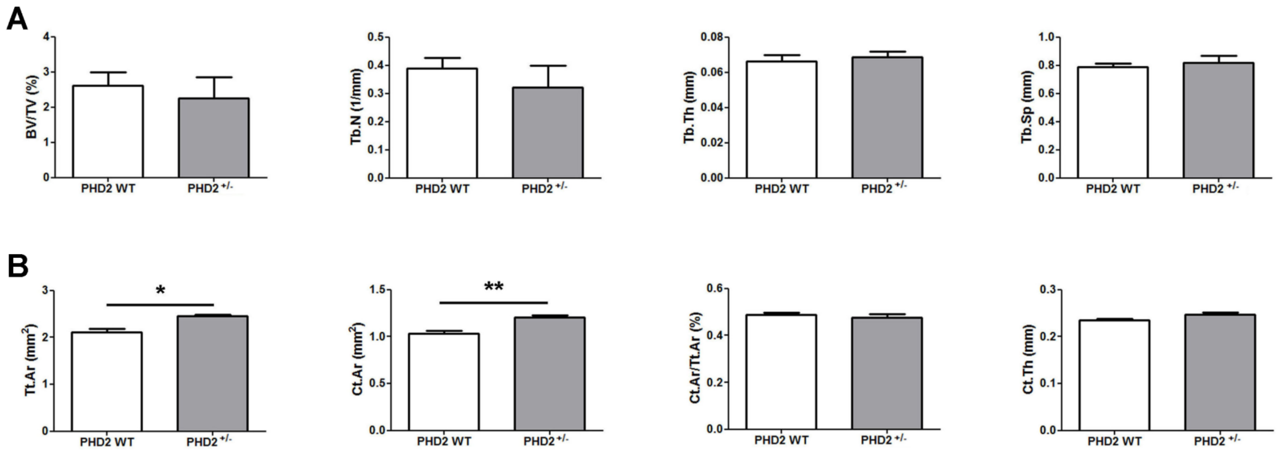


**Supplementary Figure 1. MicroCT of femoral trabecular and cortical bone from *Phd2*$^{+/-}$ and *Phd2*$^{WT}$ mice.** (a) Trabecular bone measurements; BV/TV (%), Tb.N (1/mm), Tb.Th (mm) and Tb.Sp (mm) and (b) cortical bone measurements; Tt.Ar ($mm^2$), Ct.Ar ($mm^2$), Ct.Ar/Tt.Ar (%) and Ct.Th (mm) in *Phd2*$^{+/-}$ and *Phd2*$^{WT}$ mice. *, $p<0.05$; **, $p<0.01$.
