# Supplementary material for: Hypoxia‐inducible factor 1‐alpha does not regulate osteoclastogenesis but enhances bone resorption activity via prolyl‐4‐hydroxylase 2
Source: J Pathol. 2017 May 29;242(3):322–33. doi: 10.1002/path.4906 (PMC5518186; doi:10.1002/path.4906)
Supplement: Supplementary file 2 — Figure S2. MicroCT of femoral trabecular and cortical bone from Phd3 ‐/‐and Phd3 WT mice [file PATH-242-322-s002.pdf]

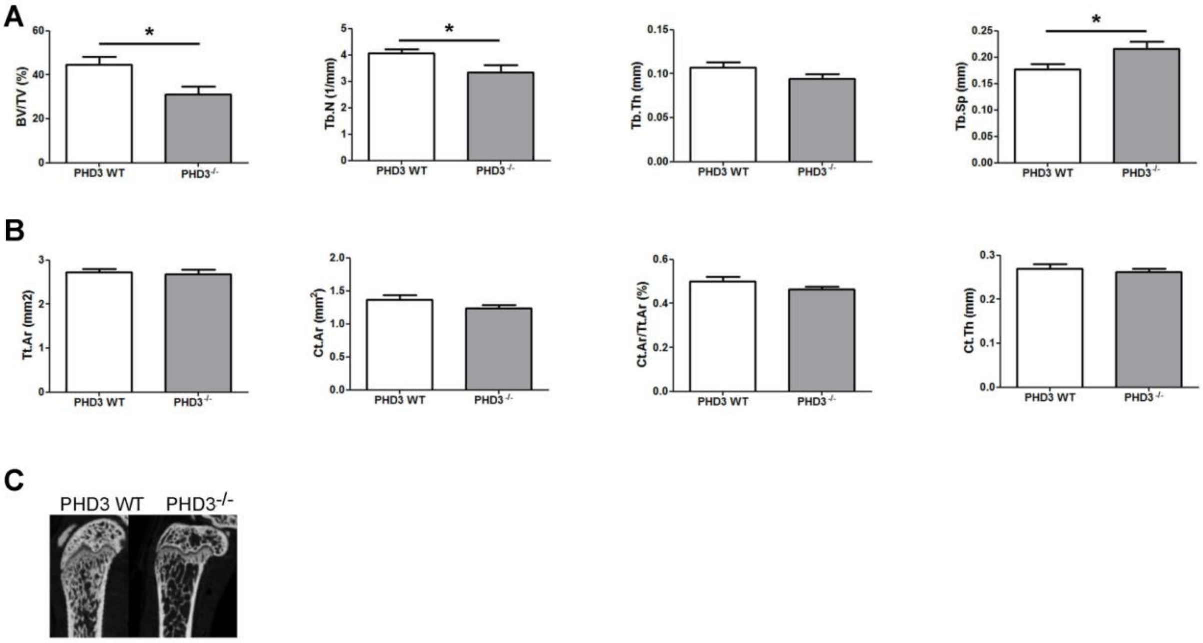

Supplementary Figure 2. MicroCT of femoral trabecular and cortical bone from *Phd3*$^{-/-}$and *Phd3*^{WT} mice. (a) Trabecular bone measurements; BV/TV (%), Tb.N (1/mm), Tb.Th (mm) and Tb.Sp (mm) and (b) cortical bone measurements; Tt.Ar (mm^2), Ct.Ar (mm^2), Ct.Ar/Tt.Ar (%) and Ct.Th (mm) in *Phd3*$^{-/-}$ and *Phd3*^{WT} mice. *, $p<0.05$. (c) Representative 2D image of femoral bone from *Phd3*$^{-/-}$ and *Phd3*^{WT} mice.
