# Supplementary material for: Hypoxia‐inducible factor 1‐alpha does not regulate osteoclastogenesis but enhances bone resorption activity via prolyl‐4‐hydroxylase 2
Source: J Pathol. 2017 May 29;242(3):322–33. doi: 10.1002/path.4906 (PMC5518186; doi:10.1002/path.4906)
Supplement: Supplementary file 3 — Table S1. List of primers used for RT‐qPCR [file PATH-242-322-s003.doc]

**Table S1. List of primers used for RT-qPCR**

|  | **Forward** | **Reverse** |
| --- | --- | --- |
| *Actb* | 5'-ATGTGGATCAGCAAGCAGGAG-3' | 5'-GTGTAAAACGCAGCTCAGTAACA-3' |
| *Angptl4* | 5'-CTTCCACTCTATCCCACGGC-3' | 5'-AGTGAGGAGGCTAAGAGGCT-3' |
| *Cebp* | 5'-GAACAGCTGAGCCGTGAACT-3' | 5'-TAGAGATCCAGCGACCCGAA-3' |
| *Ctsk* | 5'-CAGTAGCCACGCTTCCTATCC-3' | 5'-GAGACAGAGCAAAGCTCACCA-3' |
| *Lep* | 5'-TCACACACGCAGTCGGTAT-3' | 5'-AGGCTGGTGAGGACCTGTTG-3' |
| *Nfatc1* | 5'-CAGGACCCGGAGTTCGACTT-3' | 5'-AGGGTCGAGGTGACACTAGG-3' |
| *Phd2* | 5'-ATCTGGACACGAAACAAGG-3' | 5'-TCACTCACTGGCAACAATC-3' |
| *Phd3* | 5'-GCCCAGAAAGTGGCAATAAA-3' | 5'-CCAAGAAAGGGCTGGATTTT-3' |
| *Pparg* | 5'-TATTCTCAGTGGAGACCGCC-3' | 5'-ATCTTCTGGAGCACCTTGGC-3' |
| *Trap5b* | 5'-GCAGCTCCCTAGAAGATGGAT-3' | 5'-CGCCCACAGCCACAAATCTC-3' |
